# Selections and Abstracts

**Published:** 1913-03

**Authors:** 


### SELECTIONS AND ABSTRACTS

### DIAGNOSIS OF GASTRIC ULCER*

By Arthur F. Chace, A. M., M. D.

*Adjunct Professor of Medicine in the New York Post-Graduate Medical School and Hospital*

Typical gastric ulcer admits of easy diagnosis, but atypical cases present some of the most difficult problems with which we have to deal. Uncomplicated ulcers, in the same developmental stage occurring in patients of like temperament, present substanstially similar symptoms but clinically we are confronted with the task of diagnosticating gastric ulcers in all stages of development; in varying positions; and in association with other conditions such as adhesions, cholecystitis and appendicitis.

A rather detiled consideration of the classical symptoms of ulcer, together with a study of the newer methods of examination, will enable us to recognize some of the cases of ulcer which are now discovered for the first time on the operating table or at post-mortem.

Most cases of ulcer of the stomach give a history of having had for a year or more digestive disturbances which consisted

---

*Read before the Clinical Society of the New York Post-Graduate Medical School and Hospital, December 20, 1912.

of belching, acid eructations and burning sensations. They have usually had excellent appetites and have over-eaten. The taking of food relieves their epigastric distress. These symptoms have gradually progressed until the post-prandial discomfort becomes painful, so that finally, although the appetite is excellent, they are afraid to eat. The acid regurgitation increases in severity, until the patient vomits after taking food. The tongue remains moist and red. As the trouble progresses the pain becomes sharp, gnawing in character and extends to the back. It is made worse by solid food and relieved by alkalis. Pain is the most constant symptom of ulcer, existing in about 95 per cent. of the cases. Associated with the pain is marked epigastric tenderness, usually localized beneath the xiphoid to an area the size of a half dollar. Posteriorly there is also a tender area to the left of the ninth, tenth, and eleventh vertebræ. This point bears the name of Boas who first called attention to it.

Vomiting occurs in about two-thirds of the cases, coming on the first hour after meals. The vomitus consists of food, and mucus gastric juice at first, but later contains blood. Many cases have tarry stools and in practically all the cases occult blood is found chemically in the feces during the active stage of the ulcer.

Starch is usually found in the form of dextrin in test meals after ulcer; and the digestion of proteid is usually rapid owing to the increased amount of gastric juice. Hypersecretion is more often associated with ulcer than hyperchlorhydria. Over 75 per cent. of acute ulcers occurring in young people, have an increased amount of hydrochloric acid in the stomach after an Ewald test meal. Sarcinae and occult blood are often found in the gastric contents. In the chronic ulcers of older people the gastric acidity is apt to be below. From the diagnostic standpoint the tests for the motor functions of the stomach are of more value than those of the secretory. William J. Mayo believes that the finding of small particles of food in the stomach 8 to 12 hours after meals is the most important diagnostic sign of ulcer and is also an indication for surgical interference.

The thread impregnation test, devised by Dr. Einhorn, is of value not only in diagnosticating ulcer but also in locating it. Unfortunately, however, we occasionally obtain blood stains on the string in cases where we can positively rule out the possibility of ulcer.

The fluoroscope shows a break in the peristalic wave at the site of the ulcer. The X-ray is also of value in noting an adherence of bismuth to the ulcer six hours after a bismuth meal. The faintness of the bismuth shadow in small ulcers renders this method of slight value.

The use of the gastroscope in localizing ulcer is of limited value, owing to the danger attendant on its introduction. Bonninger found that the introduction of 200 c.c. decinormal solution of hydrochloric acid through a tube into the stomach, produced pain in cases of ulcer. In our hands this diagnostic sign has not been constant enough to warrant its use.

The existence of characteristic pain; vomiting; localized area of tenderness in the epigastrium and over Boas point in the back; and hypersecretion are suggestive symptoms of ulcer. The presence of blood in the vomitus or in the stools; a break in the peristaltic wave in the X-ray pictures; the presence of food particles in the stomach twelve hours after meals are confirmatory signs. It should be remembered that many cases of ulcer remain latent throughout their entire course, and as Van Valzah says, "There is no anatomical disease of the stomach, the clinical expression of which is more variable than that of gastric ulcer."

Leube wisely says: "The diagnosis can occasionally be made solely by the efficacy of the fruitlessness of a specific treatment for ulcer." A good rule is to give all doubtful cases the treatment for ulcer.

The conditions from which ulcer has to be differentiated are; gastric neuroses; cholelithiasis; gastric crisis of locomotor ataxia and cancer. One of the greatest difficulties in diagnosticating the above conditions, is the fact that gastric ulcer can exist simultaneously with any of them. Gastric neuroses may be differentiated by the irregularity of symptoms, and the absence of blood in the vomitus or stools, and the negative character of the X-ray examination. In gall-stones, the pain bears no definite relation to the meals and often radiates to the right shoulder. Tenderness over the gall-bladder and to the right of the ninth, tenth and eleventh vertebræ posteriorly, is suggestive of gall stones. Moreover in choelithiasis we are apt to have hypersensitiveness over Head's zone and other signs such as Moynihan's and Murphy's.

Each year one or two cases of locomotor ataxia with gastric crises are sent to the hospital with the diagnosis of gastric ulcer. The absence of occult blood in the stools; the negative X-ray findings; the absence of the knee-jerks; the Argyll-Robinson pupil; Romberg's symptom, and occasionally a positive Wasserman reaction clears up the diagnosis.

The differential diagnosis between early ulcers and cancer can be made by the acuteness of the symptoms and by the hypersecretion in ulcer. Cancer has a more insidious onset. Owing to the tendency of chronic ulcers to become malignant and to the impossibility of determining the exact time when this change takes place, it is advisable to operate in all cases of chronic ulcer which resist medical treatment.—*The Post-Graduate, Feb'y.* 13.

---

### DERMATOLOGY IN BERLIN

By Lloyd Warren Ketron, B. S., M. D., Baltimore, M. D.

*Special Worker in the Laboratories of Professor Ernest Finger's Clinic, Vienna.*

It would be impossible for one who has spent only one-half year in Berlin to give an accurate and extensive account of the clinics of dermatology or of those who are interested in dermatology. It would also be undesirable to attempt to give any rating of them either as to their standing as dermatologists or as to their popularity as instructors. Therefore the purpose of this paper shall be only to give a few ideas and impressions at random of the men and their work limited to those with whom I have taken courses or whose clinics I have several times visited, hoping that it may be of service as a means of orientation to those contemplating a trip to Germany for dermatological study. The first question that arises to concern the intending worker is as to where and how the most advantages may be obtained during the limited time one is permitted to remain abroad. In answer to the first part of this question one has no difficulty in deciding, for Berlin which as one of the greatest cities of Europe easily maintains itself as the center of most things medical in Germany. It makes itself especially desirable to the dermatologist because of the large number of men who are practicing dermatology in an absolutely scientific and up-to-date manner and who are not content with rapid, superficial diagnoses. Recognizing that so many diseases of the skin are merely manifestations of constitutional disorders they make the most thorough general examinations, substantiating or disapproving tentative diagnosis by clinical tests and the various cutaneous and serum reactions.

The answer to the second part of the question is one involving more consideration, all the phases of which it would be impossible to discuss. In the first place it will be at once evident that one should possess a certain knowledge of the German language, but absence of this is not such a grievous fault for it is in no way absolutely necessary to a satisfactory application of one's time. With careful study in a suitable environment a vocabulary sufficient for reading and understanding most things pertaining to one's subject can in a very short time be acquired, and in the meantime the cases which are seen should be carefully observed and recorded and points not clear read up during unemployed hours. The requirement which is probably of the most importance and upon which the successful carrying out of the above tests, is a good foundation in the rudiments and principles of one's specialty. One comes to Germany of course, to broaden his general ideas, to acquire new methods, to meet and associate with men who have attained high reputations as dermatologists, but that which is the most essential is the careful observation of cases which though common in Europe are rarely seen in America. The proper appreciation of such cases is impossible to one who must look at them all as of equal importance, and in a morning's work where perhaps thirty to forty patients are presented a rare condition may be entirely overlooked, the opportunity for observation of which may not again present itself during a short stay broad. If one is able to differentiate the material, noting carefully, of course, the common cases but making especial effort to record and remember the rare ones, he will have at his disposal a very valuable asset which will undoubtedly be of immense service in the future.

Work is obtainable in Berline, by taking special courses given by men connected both with private and public dispensaries, the only inconvenience arising when a sufficient number of men can not be found to fill them. These courses consist of demonstrations given from three to seven times weekly, lasting one month, the fee for which averages about fifteen dollars. They may deal either with the clinical, histological, cosmetic, or therapeutic side of the subject, very excellent instruction being also obtainable in light-therapy. Visiting the morning clinics is permitted but this privilege by custom is limited to a very short time. In the private polyclinic, not all of which I have had occasion to visit, one sees not so many cases as in the hospital dispensaries, but, of equal advantage, one does see many of the refinements of technic which are impossible in a large hospital clinic.

In the polyclinic of Professor Blaschko, which is very excellently equipped with the appliances of modern therapy, one has the opportunity to pick up many of the practical points of dermatology. As an illustration, in giving intra-muscular injections of mercury special emphasis is laid upon the fact, that after inserting the needle negative pressure should be made in the syringe, and that at least one minute should be waited after disconnecting the needle for the appearance of blood. In contrast to the above precautions and coming rather as a shock to one fresh from the strictly impressed rules of asepsis, is the absence of any preliminary disinfection of the site of injection, but, as no evil results have followed over twenty thousand treatments the necessity of this precaution seems excluded.

Professor Blaschko is especially known through his untiring efforts in combatting venereal disease having with Neisser founded the "Doutsche Gesellshaft zur Bekampfug der Geschlechtkrankheiten," whose honorary general secretary he now is. Through his efforts in 1897 the lepers of Northeast Germany were colonized and measures taken against the further spread of the disease. Of special interest among his works is the atlas in which he shows how the body is divided into certain segments and that some diseases of the skin as well as congenital anomalies are limited to these segments or their border lines. At present Professor Blaschko is very much interested in light therapy and his results especially with the quartz lamp have been very satisfactory.

I had many pleasant visits to the clinic of Dr. Edmund Saalfeld which has many of the conveniences that make the practice of dermatology a pleasure. An apparatus which he has invented for the application of softening agents to the face appeals to one as a very important addition to acne and comedo treatment. Dr. Saalfeld is a firm believer that cosmetics should occupy a more important place in the field of dermatology and his efforts in this line as well as his text-books are well known. In visits to Dr. Saalfeld's clinic I was especially interested in some cases of the condition first described by Kaposi-Biesiadecki as lymphangioma tuberosum multiplex, which were presented by him at a meeting of the Berlin Dermatologic Society in November, 1912. One of these which showed some clinical variation from the usual type should according to his histological observations, be classed as a true hemangioma tuberosum multiplex, i. e., having its origin in the vascular system, while the remaining ones belonged to the type which, as believed by many observers originate from the lymphatic system. Although his contentions were disputed he still believes firmly in their correctness and at present is engaged with a leading French pathologist in an attempt to clear up and establish the origin of this much-disputed condition which now has as many names as there are possibilities in the skin for its development.

Dr. Saalfeld also has made important contributions to the physiology of the skin, adding many points to the work of Langley and Sherrington on the innervation of the musculature of the hair.

A well known contributor to the field of dermatology through one of its most difficult branches is Professor Julius Heller whose text-book on Diseases of the Nails, is today one of the first authorities on the subject, and whose continual interest in this line is shown by his 1912 monographs on "Cosmetics of the Nails," and on "Some of the Rarer Nail Conditions." Professor Heller has also written an extensive work on "The Comparative Pathology of the Skin," which shows the thoroughness and completeness characteristic of the author. It deals with both the clinical and histological sides and lays special stress on the analogy existing between the diseases and congenital anomalies of man and those of the lower animals. In his laboratory are found many of the specimens used in the preparation of the above book which possess special interest to one visiting his clinic. The excellent contributions of Professor Heller to many other branches of dermatology need no new introduction.

Everyone coming to Berlin for dermatological study soon becomes acquainted with the clinic of Professor Max Joseph, which among the private dispensaries is unexcelled for its variety and wealth of material. His courses, which run continuously are given in his general consultation rooms where one sees daily all of the material thereby being able to observe their variations and progress under treatment. His style of teaching is peculiar to himself. The cases are placed one by one upon a chair and someone is requested to make a diagnosis, an error in which is not likely to be soon forgotten, as the professor uses no kid gloves in his elaborations on one's mistakes. Professor Joseph's immense amount of work shows him to be a tireless contributor to dermatological literature. His text-book on general dermatology is concise and complete as also are his special works on general cosmetic surgery, and hair cosmetics. His interest in histological pathology is well shown by his atlas which has been translated into English. Of special interest among his many researches are those on sarcoid tumors and on the experimental production of alopecia.

As to the hospital clinics, I have had occasion to visit only two, namely, the Charite and Rudolph Virchow Krankenhaus, the former of which offers the most dermatologic material. Under the supervision of Geheimrat Lesser and an able staff, it gives origin to some of the best dermatological advances in Europe. Having two well appointed buildings thoroughly equipped with laboratories, research rooms, a handsome library, and all the apparatus for light therapy, the opportunities for excellent work are all that could be desired. It is here that one sees the reward incident to being a celebrated man in medical lines in Germany. Upon the entrance of Geheimrat Lesser for his customary morning visit, chaos immediately become order and in answer to a "Guten Morgen Meine Herrn," everyone makes his best bow. The patients are immediately marshalled in a long row, stripped to the waist, and the Geheimrat with Oberartz Tomasczewski on one side and First Assistant Arndt on the other, stands at the head of the column examining the patients as his fancy may direct, at times making remarks for the benefit of a few "knowledge seekers" who are making special effort to obtain a view of the cases through the throng of assistants. After a short time the Geheimrat, with a pleasant bow to those on either side of the room takes his leave and the oppressive silence gives way to the clamor of a crowded clinic. Courses are obtainable from various assistants connected with this clinic, but my work has been limited entirely to those given by Priv.-Doz. Arndt who, though a comparatively young man, has had eight years special training with some of the best dermatologists of Italy, France, Germany and England. His teachings are not limited to the German school but, influenced by two years with Brocq and Sabouraud, involves many of the French ideas. As a histologist, he occupies a very important place in Berlin having learned, through the examination of an immense amount of material, the lesson of conservatism which is so very important in dermatological pathology. As a clinician his paintaking examination of the most simple conditions as well as his clear cut diagnosis, impress one with his thoroughness. I had the pleasure of seeing many of the illustrations for a new book which he is writing, dealing only with the diagnostic and histological side of dermatology, the latter of which will undoubtedly be especially welcomed by the profession as a very much needed addition. His extensive work on "'Der leukamischen aund aleukamischen Lymphadenose der Haut" as well as that of Sporotrichosis deserve special mention.

In the Rudolph Virchow Krankenhause the material, unfortunately for the pure dermatologist, is mostly of a genito-urinary nature, and, as the polyclinic is only in its infancy, the cases seen are mostly in the chronic class. Any disappointment, however, at a deficiency of material is more than overbalanced by the hospitality of Professor Buschke who has charge of one of the dermatological divisions. One is welcome to follow him on "morning rounds," at which time special effort is made to demonstrate cases which may show interesting features. I had the pleasure of seeing the collection of histological specimens, sent from all parts of the world, which were used in the preparation of his extensive work on Blastomycosis among which, of special interest to the American, were those sent by Dr. Gilchrist of Baltimore, one of the first authorities and a pioneer in this subject. Of much interest also is the large collection of rats and mice which he is using in the experimental production of alopecia brought about by the subcutaneous injection of thorium. These experiments have been carried on, with various modifications, for a number of years and are of peculiar interest because of the selective action of the drug, the results obtained showing various modifications in different species and a well-defined preference for certain areas on the skin of the same species. As yet no histological changes in the affected areas have been demonstrated.

The work of Professor Buschke shows the highest degree of excellence. In a hospital offering all modern equipments and a well trained crew of assistants the cases on the wards are worked up with every detail, special attention being given to any unusual deviations from normal. His contributions to Dermatology are many, their variable character showing an intimate knowledge with every branch of the subject, and his many valuable investigations, of a purely scientific nature, prove him to have devoted his life to dermatology more as a lover of science than as one merely choosing a profession.

One of course will not leave the Rudolph Virchow Krankenhaus without a visit to the division of Professor Wechselmann whose work as a dermatologist is at present lost sight of in the fierce combat he is waging as the great champion of salvarsan therapy. In his wards, which contain hundreds of syphilitics, treatment is given three or four times weekly and when one thinks of the painstaking care and elaborate technic with which salvarsan was at first administered, he scarcely believes it possible that two assistants could inject forty or fifty patients within an hour, but such is the case and the vast number, standing at the door of the treatment room, pass in and out by twos and what was formerly a day's work is accomplished now in scarcely one-tenth of the time.

The technic at present of salvarsan administrations is as follows: Each patient receives about 3 gms. which is divided into six or seven doses, the administration of which covers a period of about six weeks. The first dose is always 0.1 gm which is given as a test for any hypersensitiveness which may exist to the drug. This preliminary test is followed in two or three days by the customary dose of 0.3 to 0.6 gm. If any idiosyncrasy exists the injection is stopped immediately or continued very slowly depending upon the gravity of the symptoms Professor Wechselmann believes that most of the fatal results are occasioned through faulty technic, especially through the use of water containing the products of dead bacteria or fine particles of undissolved material. Of special interest is his explanation of certain of the so-called anaphylactic phenomena which occasionally occur in those sensitive to the drug. As these symptoms, consisting of bradycardia, dizziness and other effects of disturbed vaso-motor function, occur only after the intra-venous administration, he believes them to be due to a stimulation of the depressor nerve of the heart, holding as special confirmatory evidence the fact that they are usually preceded by a cramp-like cough which may well be due to a reflex through the superior laryngeal nerve. Neosalvarsan has been practically abandoned as results show that it is not so effective as the original product although it may be given in some cases where salvarsan is not tolerated.

One finds Professor Wechselmann very enthusiastic and, although receiving many hard knocks he still stands as an unflinching champion of his convictions that salvarsan should have its efficacy, as a specific for syphilis, thoroughly tested without the aid of mercury. He is practically the only one who is to-day using a pure salvarsan therapy and no doubt, upon the result of his many injections, which now number over twenty-five thousand, shall the fate of Ehrlich's great discovery rest.—*The Urologic and Cutaneous Review, Feb'y.,* 1913.

---

### THE DIAGNOSTIC SIGNIFICANCE OF PAIN IN TABES

By Allan McLane Hamilton, M. D., LL. D., F. R. S. (Edin), New York

Tabetics in the early stage of their disease, as a rule, complain of the familiar pains situated mostly in the lower extremities, and in the apparent absence of qualifying symptoms these are often mistaken for other forms of disease; in fact the ordinary patient with locomotor ataxia frequently insists in the beginning that he has had rheumatism or neuralgia. The similarity of pain, due to irritation of the posterior nerve roots, with that of the more common disease, is sometimes further increased by the influence of barometric changes and enlargement of the joints, which in the spinal affection constitutes the neuroosteorthropathies. While it is true that the latter joint troubles are rarely painful, this is not invariably the case, and there is sometimes room for great error.

Early tabes is therefore by no means so easily diagnosticated as may be supposed, because of what Fournier* calls

*Alfred Fournier, De Pataxie locomatrice d'origine syphlitque, Paris, 1882.

the initial polymorphism," which is expressed by symptoms common to, and often mistaken for those of other affections. Among these are the peculiar access of fulgurating pains, which are not always as characteristic and definite as some medical writers aver, gastric crises, ocular paralysis, failure of vision and inability to discriminate colors, vesical disturbance, laryngeal spasm, and a number of other preataxic manifestations.

The pains, however, are the most important indication of the commencing sclerosis of the posterior nerve roots, and may be of very early appearance, constituting the only dominant or even apparent symptom for perhaps even ten or twelve years; but I doubt if there are many cases where some of the lost reflexes may not at the same time also be detected, especially after a year or two. The severity of the peripheral pains, however, does not equal the so-called abdominal or thoracic crises which symptomatize tabes and are erroneously attributed to other conditions, often leading to improper medical treatment or surgical interference. The likelihood of error is also increased by the fact that these are of early appearance in one-third of all cases of tabes, and they may be uncomplicated for years.

Charcot first described the so-called crises gastriques as pains beginning in the groins and running up the abdomen on either side, finally becoming fixed at the epigastrium.**

**A very early reference was made by Romberg to these pains in his classical work, A Manual of the Nervous Diseases of Man, Sydenham translation, ii, p. 397.

This description is not an altogether broad one, for the erratic nature of the pain is characterized by variations in its appearance, and sometimes it may be accompanied by violent palpitation, vertigo, and vomiting, but these features are not always present. Like the fulgurating pains of the lower extremities, notably in the muscles supplied by the crural nerves, they are manifested by patients who are subject to osteoarthritic changes. Buzzard presented six patients with arthropathy of whom all had crises, and this is the experience of others. The pains, which are more commonly epigastric than of any other variety, differ greatly, not only in their mode of onset and disappearance, but in their relation to the condition of the stomach itself. At the risk of repeating much that is well known, passing reference may be made to the three varieties of gastric pain fixed by Vulpian, Charcot, and Fournier, viz.: 1, Gastric colic (or the grand gastric crises of tabes); 2, vomiting with pain; 3, gastralgia alone.

These seizures do not always occur at the time of eating, although the pain at first often seems to be due to gastric indigestion and there is intolerance of everything taken, even of ice, which is afterward ejected in the form of warm water. The attacks of pain become almost habitual, and the vomiting lasts perhaps for five or six days. The latter may be "dry," or it consists of mucus and perhaps some blood. The true gastric colic has an absolutely sudden appearance, and the acute pain may be localized. Sometimes it radiates to back, thorax, or abdomen; again it follows the course of the intercostal nerves. Not only are epigastric pains familiar, but Raynaud speaks of renal neuralgia, and Stewart of deep seated dorsal and lumbar pains. With the tabetic renal neuralgia there are even at times vesical tenesmus and retraction of the testicle. Bearing in mind the very extensive supply of nerves with sensory functions it is possible to conceive of almost any kind of pain other than those ordinarily known and looked for. Indeed, pain at crisis may counterfeit that from local disease of numerous kinds, and we must be prepared sometimes to disregard the positive qualification that it is invariable of sudden appearance and departure. In some tabetics the attacks are so closely connected and apparently so continuous as to convey the idea that they have nothing to do with a spinal lesion. I have known tabetic rectal neuralgias to be mistaken for the sharp pain of cancer, stricture, fissure, or other conditions, and the diagnosis adhered to until an unsuccessful operation had been performed. Such a case is one where a stricture of the anus was alleged to exist, and repeated stretching was resorted to without any result whatever, but the pain was ultimately found to be due to genuine locomotor ataxia.

Another case of this kind was recently seen in consultation. According to this patient's story, about five years ago he consulted a surgeon for rectal neuralgia, who removed hemorrhoids, but with no resulting relief of the pain, which was described as exceedingly violent, burning, and paroxysmal in character, darting chiefly toward the perineum. He subsequently consulted several surgeons, one of whom removed a testicle, while another made a deep posterior incision, cutting through the sphincter ani, but these operations were inefficacious. He finally fell into the hands of an intelligent medical man whose attention was drawn to a deep cup shaped erosion on the ventral side of the glans penis. This was the site of an old venereal sore, supposed to be a chancroid, which had followed an infection when he was a boy of fifteen years. The man is now about forty. Ten years ago he became suddenly deaf in both ears, but chiefly on the right side. About one year ago he had, according to his story, a transient attack of ocular muscular paralysis so that he had to correct his diplopia when reading by changing the position of the book before him. It is impossible to say exactly of what this consisted, though there is to-day an apparent slight internal strabismus. In March, 1912, according to Dr. David Geringer, he was "mentally deficient, indifferent, and melancholic." At the same time there was a marked Romberg sign, and the pupils were then both rigid. According to Dr. J. W. Weeks, who then saw him, he "had a tendency to Argyll Robertson pupils." A Wassermann test was made in May, 1912, which was weakly positive. He then received 0.6 gramme salvarsan by intramuscular injection, and one month later a second test proved negative. The intravenous neosalvarsan injections of which he subsequently received three—0.6, 0.9 and 0.9 gramme—did not modify his pains, which were intense, but "seemed to improve him physically."

I saw him prior to an operation undertaken by Dr. A. B. Johnson for the repair of his mutilated anus and rectum, on

November 21, 1912. He immediately said he had a violent crisis, referred to his anus and entire perineum and pain which caused him to draw away and place his hands over the lower part of the abdomen. This pain often occurred with intensified desire to urinate, and a feeling as if an orgasm was to take place. While there was an emotional element, suggesting hysteria, there could be no doubt of the spinal origin of the painful paroxysm. He referred to fulgurating pains in the thighs of more remote occurrence. Argyll Robertson pupils were found, but the knee jerks and Achilles reflexes were greatly exaggerated as were the plantar reflexes. There was some static ataxia, but no motor ataxia. There was neither tactile anesthesia nor analgesia, but, on the right side especially, the thermic sense was disordered. No Babinski symptom was elicited. There is no difficulty in voiding urine or retaining it, but owing to the uselessness of the sphineter ani, patient constantly loses the contents of the lower bowels.

Crural neuralgia of an explosive type is a tabetic symptom, and when it is associated with retraction of the testicle it has led to the delusion that a calculus existed. It is unusual, however, to find pain at the end of the penis, which is a symptom of nephritic colic. The crises suggest involvement of the branches of the lumbar and sacral plexus in the groin, thigh, and hypogastrium. There is rarely scanty or high colored urine, in fact in the tabetic condition the distress is accompanied by a copious and repeated flow of urine. As the sudden liberation and passage of a stone through the ureter is attended by sudden cessation of suffering, such may be the case in the pseulocolic due to the spinal lesion. In many cases of nephritic colic, I have found spots of residual tenderness, not only in the lumbar, but in the hypogastric regions as well, and the same thing in hepatic colic over the region of the gallbladder and common duct. My attention has been called to a recent case in which an operation for gallstones was happily averted despite the insistence of the attending physician that the gallbladder should be removed. The medical man who was called in was a competent neurologist, who promptly discovered rigid pupils and other evidences of a long standing tabes without any other dominant symptoms. The diagnosis was made more interesting because of the fact that the patient was a women, and, as less than two per cent. of all tabetics are of this sex, an additional doubt was raised. In one of my own cases, in a remarkably slowly progressive case, the same thing occurred. Finally, distinct gastric crises made the case more clear, and with this the absence of knee jerks became evident.

Starr refers to a tabetic patient in whom gastric crises existed, and the attacks reappeared at intervals of three or four months for four years before loss of knee jerks and Argyll Robertson pupils were found and made their nature clear. The patient meanwhile had been treated by lavage, special diet, and in other ways with the idea that the condition was simply a gastric one. Most writers have something to say about the errors in diagnosis that have been made in cases of simple ulcer, in fact any gastric condition characterized by ephemeral pain of sudden appearance, with or without nausea or vomiting, may be confounded with the pain of the more serious diseases.

S. Basch,** in an exceedingly valuable article upon this subject, refers particularly to the distinction between gastric crises and the pain of ulcer or cancer of the stomach, perigastritis, gastrosuccorrhea, functional nervous vomiting, gastralgia, duodenal ulcer, cholelithiasis, cerebral vomiting, and vomiting of uremia. I do not think we need concern ourselves with the latter two conditions although they may at times suggest the central nervous disease, but the important of mistaking the pain and vomiting of gastric ulcer cannot be minimized. Basch calls attention to the fact that the pain of gastric ulcer begins usually half an hour after eating, and is situated at a fixed point in the epigastrium. Then, too, there is a coincidence between the time of ingestion of food and the vomiting, "the latter taking place at the height of digestion." The vomited matter as the result of ulcer usually contains free hydrochloric acid and blood, the former in large quantities. In gastric ulcer the pain is as a rule gnawing as it is in cancer. Osler speaks of a

**S. Basch, Medical Record, October 14, 1899.

form of epigastric pain which is found in ulcer of the stomach at about the level of the tenth dorsal vertebra.

The appearance of tabetic gastric pain is not always in the classical manner referred to by Charcot, but may have existed, as I have said, for years without any other symptom. In some rare examples, one pupil only may at first have become rigid, but not markedly so, and it can be conceived how a careless examination which does not include both eyes may lead to error. A perimetric examination will also show in certain cases a contraction of the field of vision for the perception of both colors and white. Reference may be made to a case of doubtful tabes reported by Brudenell-Carter, with slight central impairment of the vision of one eye, and a tendency to pallor of the optic disc; as a rule, however, the fields of both eyes are narrowed. In a lesion of the kind leading to delayed reflex loss, it is probable that the lesion in the posterior columns is circumscribed, small, and confined perhaps more to the root zones of the columns of Burdach and possibly to the lumbar region. The deep pains are the most apt to deceive the investigator, for superficial sensory disturbance, paresthesia, and the like are too characteristic to be mistaken.

The pain of supposed aortic disease is sometimes very intense and may have some explanation than that generally adopted. So called angina pectoris, if occurring in comparatively young subjects, need not depend upon aortitis or serious valvular trouble, stenosis, or regurgitation. When the sudden pain is disconnected from undue muscular exertion, or ever occurs during sleep, the gastralgia may be due to unrecognized tabes; in fact, in over six per cent. of the cases of tabes there is some aortic disease, the actual cardiac lesion being due to local syphilis. In many other cases with no arterial or valvular lesions there may be crises of pain which are of purely central origin, the individual being tabetic. Even in properly diagnosticated thoracic aneurysm the pain may sometimes be the sole result of posterior spinal sclerosis, as Clifford Albutt has pointed out. The clinician should never avoid a search for the condition of the reflexes in every case of dorsal or extremity pain, no matter how clear the diagnosis may seem at first, and the patient should be examined with regard, not only to the response of the pupil to direct illumination, but to accommodation, though Argyll Robertson's symptom is of not very early appearance unless the disease at its invasion attacks the upper dorsal segments, which is unusual. It may be present long before there is any pain or ataxia. In my own cases, I long ago** reported the early presence of diplopia, even without apparent strabismus. These ocular symptoms as a rule come later than the loss of the knee jerk which obviously depends upon a lesion involving the second and third lumbar segments, this being the early region of invasion.

**Allan McLane Hamilton: Nervous Diseases. Their Description and Treatment, Philadelphia, 1878.

Two forms of occasional pain may resemble those due to disease of the posterior columns, and these are the cramplike abdominal pains symptomatic of arteriosclerosis of the big mesenteric artery, and the intermittent claudication due to arteriosclerosis, as well as to tobacco toxemia. In the former the duration of the pain is not so protracted nor is the pain so intense as in intrue tabes, while the tonic contraction in claudication is the chief feature, and the distress nearly always confined to the posterior leg muscles. There are anomalous cases, however, when the pains are most extensive in the course of the crual nerve and the muscles it supplies.

Various hidden and serious arterial disorders may for a very short time deceive the physician, but there is inevitably a clearing up with revelation of the real nature of the condition. I can remember such a case in the person of a patient whose pupils had been rigid for some time, although she presented no other sign of tabes, but had symptoms of advanced thyroid disease, the pupillary contraction being evidently due to an anomalous sympathetic irritation instead of the well known paresis and dilation usually found. When shopping one day, she was suddenly seized with agonizing abdominal pain and vomiting, and, some hours afterward, by moderate tympanites, and later by obvious intestinal obstruction. There was at first no apparent gastric cause and the association of the pain with the pupillary rigidity suggested a true crisis. There were, however, features suggesting something more serious, and the diagnosis of embolism of the mesenteric artery was made by the surgeon who was called in.

In all cases of trunk pain of doubtful nature, an effort should be made to investigate a possible history of earlier sensory disturbance, which in true tabes involves the lower extremities. By many clinicians the distinctive character of the fulgurating pains of the lower extremities is considered to be sufficient without the verifying accompaniment of lost pupillary reflexes and absent knee jerks. Segun,** who was a careful observer, even went so far as to say that fulgurating pains in all men over twenty years of age were of tabetic origin or at least "very suspicious." The very vagrant nature of these cannot be confounded with the pain of gout, rheumatism, or neuralgia, although the twinges of the former resemble the shooting pains of locomotor ataxia. The extreme limitation of the painful regions is significant, for the areas are circumscribed and circular or elliptical or they radiate from some central point. Again, the darting streak of pain is short, and does not follow the course of a nerve trunk; the painful points that remain may be at any cutaneous region and differ from those of neuralgia when the tenderness is found over some foramen or point of escape of the nerve itself. In the recurrence of pain the intervals are very short, suggesting the alternation of a galvanic current than the continuity ot the direct.

**Boston Medical and Surgical Journal, December 25, 1890.

Although modern surgery has at times afforded some relief from the suffering attendant upon gastric crises, the results are not always encouraging, and the pathological explanation of this symptom is still a matter of doubt, consequently the exact diagnosis is difficult. It is by no means clear whether the pneumogastric is at fault, or whether the pain is due only to some condition relievable by section of the spinal nerve roots. Vallas and Cotte, in 1906, opened the abdomen, and after freeing the solar plexus from the celiac axis, stretched the same; relief was asserted. By these reporters and others the sensory fibres from the pneumogastric and sympathetic system are supposed to be at fault in such conditions. On the other hand, Head believes that the sensory fibres innervating the stomach are derived from the seventh to the tenth dorsal roots, and Frazier*

*American Journal of the Medical Sciences, January, 1913.

has repeatedly performed rhizotomy severing the seventh, eighth, ninth, and tenth sensory roots, with relief in all his cases. In one of my patients operated upon at Johns Hopkins Hospital extensive section of all these nerves, however, failed to afford any permanent benefit and the patient finally succumbed to the great agony of his pain and consequently prostration after his return to his home some years later. This case would point to the pneumogastric origin of the crisis.

So far as I know, no deep operation has been devised for the relief of rectal crises, for the sacral nerves are not only inaccessible, but, if it were possible to reach and divide them, much more serious mischief would be the result.—*New York Medical Journal, Feb'y. 22, 1913.*

---

### NEPHRITIC HYPERTENSION*

*(Clinical and Experimental Studies)*

By Theodore C. Janeway, M. D., New York
*Professor, Practice of Medicine, Columbia University*

Seventy-five years ago Richard Bright first published his observations upon the relation of hypertrophy of the heart to disease of the kidneys. His work was a combination of most careful and painstaking clinical observation with subsequent post mortem examination wherever possible. He showed that in the greater number of patients with cardiac hypertrophy there were definite lesions in the kidneys. He showed further however, that cardiac hypertrophy did occur in patients whose kidneys were to all appearances normal. Bright did not indulge in fanciful speculation, but contented himself with the statement of observed facts. Those who have come after him in the study of this subject have supplied the speculative and fanciful interpretations of which we hear so much.

It was only after the perfection of the Riva-Rocci sphygmomanometer that the relation of high blood pressure to the presence of cardiac hypertrophy and renal disease was observed and subjected to study. And it has been only within the past twelve years, since a number of life insurance companies have made blood pressure readings a matter of routine, that we have had a really correct index of the normal and abnormal limits of this pressure. We may now safely consider a blood pressure above 160 mm. Hg. as abnormal and, in all probability, more extended records will somewhat lower this figure.

In the following presentation I will use the term "renal disease" in the broad sense and not attempt to limit the expression "nephritic hypertension" to any single anatomical type of renal lesion.

Among the first of the theories offered to explain the occurrence of hypertension and cardiac hypertrophy was that of reduction in the renal circulation through disease involving the glomeruli. This seemed, at first sight, an adequate explanation for those cases in which considerable disease of the kidneys was found, but it failed to account for those in which there was little or no renal involvement. Further, it was shown that partial ligature of both renal arteries did not cause any material alteration in the systemic blood pressure. The same negative results were obtained in the experimental production of thrombosis of the renal vessels by the injection of paraffin. Other workers approached the problem by removal of one kidney in dogs and then caused graded reduction in the size of the other by successive excisions of portions. In a long series of such experiments five dogs showed an average rise in blood pressure of 21.5 mm. Hg. together with some hypertrophy of the left ventricle of the heart. I have recently conducted experiments on dogs along similar lines, excising one kidney and ligating some of the branches of the renal artery at the hilum of the other, thus causing infaction and reduction in the total renal tissue of the animal. In two dogs I obtained prolonged rise in blood pressure of a moderate grade, combined with albuminuria and polyuria.

As a result of such observations on the reduction of total kidney volume in dogs there are three constant changes which have to be explained:

1. Vascular hypertonus.
2. Hypertrophy of the left ventricle.
3. Hypertrophy of the left auricle and right ventricle.

The first is now recognized as a constant phenomenon secondary to such reduction and has not been explained. The second is held to be secondary to the continued hypertension induced by the hypertonus of the systemic vessels. The third is in turn secondary to the development of stasis through partial failure of the hypertrophied left ventricle.

To account for the increased tonus of the systemic vessels seen in these experimental studies and in clinical cases in man, two theoris have been offered. The first is based upon assumption of an increased viscosity of the blood. This is little more than a theory, as it is unsupported by either experimental or clinical observations and, in case of the greatest increase in blood viscosity, such as extreme polycythemia, high blood pressure is often absent.

The second hypothesis is the more important. It predicates the existence in the blood of chemical bodies which cause a stimulation of the vasoconstrictor mechanism. This hypothesis affords ground for study along a number of lines. Two chief theries have been suggested, namely, first, that there is some accumulated pressor substance, probably arising from renal autolysis, constantly thrown into the blood stream; the second, that there is an increase in the epinephrine content of the blood.

As to the first hypothesis, we may state that the evidence is variable and very conflicting and, on the whole, is not convincing. One of the most important objections that can be raised from the clinical point of view is that hypertension is the greatest in those cases which are the most chronic and it is precisely these in which the autolysis of the kidneys is the least marked.

The epinephrine theory was first propounded by Neusser, who assumed an increased activity of the suprarenals due to a hyperplasia of the glands. He reported several cases in which this hyperplasia could be demonstrated, but it has been shown that such an occurrence is rare and that in other cases there may be a distinct hyperplasia without any increase in the blood pressure. Experimentally it has also been shown, in technically perfect operations, that complete exclusion of the suprarenals from the circulation is not associated with a fall in the blood pressure. Many observers, however, contended that even in the absence of any hypertrophy of the adrenal glands there might be an increased production of epinephrine. They sought to prove their point by determining the amount of blood pressure raising substance present in the blood taken from patients showing hypertension and comparing this with results obtained with the blood from normal individuals. Results were again conflicting, some reporting a greater vasoconstriction from the blood in the hypertensive cases, others reporting no difference. Those who found the blood from the hypertensive patients more active held that this activity was due to an increased epinephrine content, and, therefore, regarded this as confirming the epinephrine hypothesis. The careful work by O'Connor showed the fallacy of these contentions by the demonstration that epinephrine would cause an inhibition of the movements of the excised and perfused living intestine, while the substance found in the blood in the hypertensive cases produced the opposite effect, although it did cause vasoconstriction. He further showed this pressor substance could be found only in blood which has been clotted, and that if clotting is prevented by citrate or hirudin no such substance is demonstrable.

A further test for the presence of epinephrine is its power to cause dilation of the coronaries when perfused through them. Applying this test, I have been able to confirm the findings of O'Connor, showing that the substance which caused vasoconstriction of the carotid artery also constricted the coronaries, and was therefore, not epinephrine. I further found, with O'Connor, that this pressor substance is to be found only in blood which has undergone clotting. It may, therefore, be regarded as resulting in some way from the chemical processes which the blood undergoes in clotting and not as epinephrine.

In unclotted blood from hypertensive patients I have been unable to find any trace of epinephrine using the following biological tests: 1. The perfusion of the fresh carotid artery of the ox; 2, perfusion of the coronaries; 3, perfusion of a loop of living intestine; 4, the frog's eye of Meltzer.

The study of the epinephrine content of the blood in hypertensive patients may be approached from one other side. It has been shown that in animals whose livers are well stored with glycogen the administration of epinephrine will cause a hyperglychemia. Now in one hypertensive patient at present under my care we have found a hyperglychemia. The condensed table of observations is given:

| Date. | Diet. | Blood sugar. | Blood pressue. | Urea con centration |
|---|---|---|---|---|
| November 25... | NaCl poor | 0.099 | 235 | 0.168 |
| December 20.... | NaCl poor | 0 133 | 220 | ..... |
| x January 18.... | each 2 hours after | 0.201 | 230 | 0,188 |
| x January 22.... | 100 gm. glucose | 0.192 | 225 | ..... |
| x February 8.... | N. poor | 0·1605 | 205 | 0.375 |

A noncritical analysis of these findings seems to offer strong support to the view that the hyperglychemia in this case offered an index of the presence of an excess of epinephrine in the blood and that this was, therefore, the cause of the hypertension. Critical inspection, however, shows that there is no relation between the blood sugar concentration and the blood pressure, and that the two are independent phenomena. The explanation suggested is the retention by the kidneys of toxic products of metabolism, and this is strongly supported by the extremely high urea concentration found. At the times marked x the blood was tested for epinephrine and, while some of the tests were very suggestive, in no single specimen was I able to secure positive results by three of the four methods used, although two were often positive.

In view of these confusing results in the epinephrine tests, I gave a dog a constant injection of a known amount of epinephrine and took blood one minute before the cessation of the injection, and while the blood pressure was still high. The results lead me to the statement that no proof of the presence of epinephrine in the blood is sufficient unless at least three different methods of testing give positive results.

From these and other observations we may conclude that it cannot be assumed that epinephrine exists in the blood of hypertensive patients in sufficient amounts to maintain high blood pressure.

Basing my remarks upon my observation of 459 patients with blood pressures above 160 mm. Hg., I would group them into two classes:

1. Hypertensive cardiovascular cases.

2. Hypertensive cases with cerebral symptoms.

The first group shows disease of the renal arterioles, but there is reason to believe that this may be secondary to a maintained functional hypertonus and hypertension. In addition, patients frequently show arteriosclerosis and cardiac hypertrophy, and they die from apoplexy. In my opinion the hypertonus has its origin elsewhere than in the kidneys in some factor which disturbs the vasomotor regulatory mechanism. With these cases we certainly see the association of gout, and, in some instances, dietetic and hygienic measures seem to give prolonged relief in the earlier stages, showing conclusively that, then at least, the hypertension is functional and not due to organic change in the vessels. We know of only one definite cause of this functional hypertension, that is lead. In addition to what has been said, I believe that we must consider renal retention as a cause in a number of the cases. This is supported by experiments already cited and by clinical observations in cases of total ureteral obstruction by cancerous growth. In the later stages of this type the development of chronic passive congestion may lead to the secondary renal changes so often found at autopsy.

The second group is of equally obscure etiology, but it presents a different picture. These patients usually show polyuria and in them uremic symptoms are prone to develop.

In support of the retention theory I may cite my own observations, in which I have seen transitory hypertension in cases of acute nephritis.

Lastly, let me urge that in the present state of our knowledge it were wise to treat these cases of both groups from a purely clinical standpoint rather than in the light of present renal pathological anatomy.

The necessity for further experimental work along these lines is patent to all, but it is my belief that we must also resort again to the methods of Richard Bright, observe with exquisite care both at the bedside and in the post mortem room, and confine our statement to fact, leaving theory alone as likely to confuse the issue.—*New York Medical Journal.*

---

### THE AMERICAN ASSOCIATION OF ORIFICIAL SURGEONS

"The spring clinic of the American Association of Orificial Surgeons will be held in the Surgical Amphitheatre of Hering Medical College, corner Wood and York Sts., Chicago, Ill., April 23-4-5-6. Dr. E. H. Pratt, A. M., M. D., LL. D. and assistants will operate on clinical patients, demonstrating the fundamental principles of Orificial Surgery as applied in the treatment of chronic diseases and as an adjunct to major surgery in general

On April 26th, the fourth and last day of the clinic, Dr. Pratt and assistants will demonstrate other therapeutic measures which have been recently introduced to the medical profession; including abdominal calisthenics, manual therapeutics, high frequency treatment of internal organs, spondylotherapy and new hydro-therapeutic measures. These measures will be introduced and demonstrated not as curative measures within themselves alone, but as adjuncts to the ordinary armamentarium of the physician.

Tuition to this clinical course is free to all practicing physicians, medical students and nurses.

Physicians are invited to bring clinical cases for operation. No operating fee will be charged. Excellent hospital accommodations will be provided. Opportunity will be presented for the physicians bringing clinical cases to assist personally in the operation.

The clinic headquarters will be the Hotel La Salle where reservations may be made in advance. For further information address the Secretary of the Association,

W. A. Guild, Des Moines, Iowa.

---

### MISAPPREHENSION AS TO THE NOVELTY OF THE FRIEDMANN TREATMENT

In view of the newspaper sensation caused by the announcement by Dr. Friedmann of another "cure" for tuberculosis, it would seem well to review some of the facts previously known. Even editors of medical journals, who should be conversant with the history of medicine and of tuberculosis, have been led into grave error. For example, a recent editorial in a medical journal, from which the Literary Digest quotes, makes the statement that "Friedmann came to the conviction that the most potent curative and immunizing powers lie in the living bacterial organism itself, and not in the dead organism as used in the method of Wright and his school." The editorial says further, "Our own impression from the entire debate is that Friendmann has enunciated a principle of far-reaching consequence and his probably discovered a remedy that influences tuberculosis favorably." The effect of this editorial is to credit to Friedmann the discovery that in order to produce immunization against tuberculosis living cultures are necessary. For the truth of medical history as well as the credit of American physicians it is well to point out some of the things which were done years ago.

In 1892 and 1893, Trudeau of Saranac Lake demonstrated the fact that subcutaneous inoculation of living cultures of the avian tubercle bacillus greatly increased the resistance of rabbits against infection by virulent mammalian cultures. He immunized rabbits to such an extent that when they were inoculated with virulent cultures, the inflammatory reaction gradually disappeared, leaving the eye in the normal condition, while in control animals the destruction of the eye was complete. As far as we have been able to discover, Trudeau was the first to announce the principle that living cultures must be used in order to produce an efficient immunity against tuberculosis.

De Schweinitz in 1894 immunized animals with human tubercle bacilli which had been cultivated for twenty generations on slightly acid broth. At the end of this time the cultures were not virulent for guinea-pigs, but were capable of immunizing these animals to such an extent that they resisted infection with the bovine germ. Control animals died in seven weeks.

Pearson and Gilliland demonstrated that human tubercle bacilli which were not virulent for cattle would produce a high degree of immunity when injected into the circulation. In 1905 the same authors demonstrated a strong curative action on tuberculosis from injections of non-virulent tubercle bacilli derived from human beings.

Webb and Williams demonstrated that immunity against tuberculosis could be produced by inoculation of living tubercle bacilli, beginning with small doses and gradually increasing.

If we go to foreign publications it is easy to multiply instances of the use of living cultures. In 1901 McFadyean not only demonstrated his ability to produce immunity in cattle by the use of living cultures, but also in one case treated an animal which was already tuberculous. The animals resisted for a long time injections of tubercle bacilli of proved virulence for cattle.

In 1901 von Behring announced his method of bovovaccination, the first detailed publication of which appeared in 1902. Living cultures were used.

In 1903 Thomassen reported experiments in which by the intravenous injection of human tubercle bacilli into young cattle be produced a considerable degree of immunity.

Vallee reported experiments in which young animals were rendered highly immune against virulent bovine infection by the use of non-virulent living cultures derived in the first instance from a horse.

Instances could be multiplied, but these are enough to demonstrate that Friedmann has not discovered or announced any new principle in regard to the immunization against tuberculosis. As far as our knowledge goes, he has followed methods which were demonstrated first in this country, and have been confirmed by many workers in America and Europe.

The psychology of the excitement of the public over Friedmann is hard to understand except on the basis of clever press-agent work. Practically every fact which he has brought forward has been known for years. Why the bacillus from the turtle should possess special curative value for the human being is a mystery, although, of course, it cannot be denied that it is within the range of possibility that such a thing may be true. The point of scientific interest that should be made clear, however, is that he has discovered no new principle, at least as far as the published communications go. The principle of using slightly virulent cultures derived from another species were demonstrated by Trudeau in 1891 and 1892. The principle of intravenous injection for the best production of immunity against tuberculosis was demonstrated by Pearson and Gilliland and others in 1902. It seems, then, that if he has discovered anything at all it is only a culture which possesses unusual immunizing powers for human beings.

Practically, of course, the point of interest is in the question whether or not a harmless and clinically efficient immunizing culture has actually been worked out. On this point we still await authoritative tests, for we have no information that Dr. Friedmann has yet submitted his treatment to investigation by competent and unprejudiced experts in the treatment of tuberculosis. The announcement that he has agreed to submit his method to be tested by the New York City Department of Health and by the Public Health Service will, perhaps, be viewed with natural skepticism until the test has actually taken place, in view of the fact that he withdrew the offer to submit the treatment to his own government. If it proves its worth under adequate, unbiased scientific investigation, the medical profession, of course, will be only too glad to forget the unfortunate features in its exploitation which have raised a presumption against its worth. Until then, the public and the profession alike may be pardoned for remembering that a patent was applied for and the treatment advertised before its value was established; that as yet we have the word of no one who has actually tested it except Dr. Friedmann and his assistants or associates and, finally, that although there is no lack of clinical material in Germany, he has chosen to bring it to America first from no other apparent motive than pure commercialism.—*Journal, A. M. A., March* 8, 1913.

